# Development and validation of a machine learning predictive model for one-month post-revascularization angina in patients who had undergone PCI or CABG

**DOI:** 10.3389/fcvm.2026.1747832

**Published:** 2026-02-13

**Authors:** Jincheng Wang, Conghui Zhou, Bihua Tang, Jingqing Hu

**Affiliations:** 1 Institute of Literature in Chinese Medicine, Nanjing University of Chinese Medicine, Nanjing, China; 2 Affiliated Hospital of Integrated Traditional Chinese and Western Medicine, Nanjing University of Chinese Medicine, Nanjing, China; 3 Chinese Medical College, Tianjin University of Traditional Chinese Medicine, Tianjin, China

**Keywords:** angina recurrence, machine learning, post-revascularization angina (PRA), predictive model, risk prediction

## Abstract

**Background:**

Recurrent angina pectoris following coronary revascularization via percutaneous coronary intervention (PCI) or coronary artery bypass grafting (CABG) poses significant clinical challenges, associated with reduced quality of life and increased healthcare burden. Traditional risk tools have limitations in predicting short-term recurrence. This study aimed to develop and validate a machine learning (ML) predictive model for post-revascularization angina (PRA).

**Methods:**

This study used patient data from 38 clinical research centers in 23 provinces of China from 2016 to 2018. Data from 626 patients in a derivation cohort recruited from 28 centers across 16 Chinese provinces and 127 in an external validation cohort from another 10 centers across 10 provinces were analyzed. The Boruta algorithm selected key features, and eight ML models were trained on 70% of the derivation cohort, internally validated on 30%, and externally validated. Performance metrics included area under the curve (AUC), decision curve analysis (DCA), accuracy, sensitivity, specificity, and F1 score. The Shapley Additive explanation (SHAP) values provided model interpretability.

**Results:**

The Boruta algorithm selected six features: New York Heart Association (NYHA) classification, cardiac troponin T (cTnT), prothrombin time (PT), depression severity, abdominal circumference, and diastolic blood pressure (DBP). The Random forest (RF) model outperformed others, achieving an AUC of 0.90 (accuracy 0.88, sensitivity 0.77, specificity 0.92, F1 0.78) in internal validation and 0.87 in external validation. The SHAP algorithm confirmed the features’ predictive importance, with higher NYHA class, elevated cTnT, and depression severity positively influencing PRA risk.

**Conclusions:**

This RF model offers a robust, interpretable tool for early PRA risk stratification, integrating cardiac, hemostatic, psychological, and metabolic factors. It supports personalized post-revascularization care, though prospective, multi-ethnic validation is needed to enhance generalizability.

## Introduction

Coronary artery disease (CAD) remains one of the leading causes of morbidity and mortality globally, affecting millions of individuals and imposing a substantial burden on healthcare systems (1). Revascularization procedures, such as percutaneous coronary intervention (PCI) and coronary artery bypass grafting (CABG), are cornerstone therapies for restoring myocardial perfusion in patients with symptomatic CAD, particularly those with unstable angina or acute coronary syndromes (2). Despite advances in procedural techniques and adjunctive pharmacotherapy, post-revascularization complications, including recurrent angina pectoris, continue to pose significant clinical challenges (3). Recurrent angina, defined as the re-emergence of chest pain due to myocardial ischemia, can occur in the early postoperative period and is associated with reduced quality of life, increased hospital readmissions, and higher healthcare costs (4).

The incidence of angina recurrence following revascularization varies depending on the procedure type and patient-specific factors. Studies have reported that up to 35.4% of patients experience recurrent angina within the first year after successful CABG, often attributed to technical issues, graft failure, or progression of native vessel disease (5). In contrast, PCI, while less invasive, is associated with higher rates of target vessel revascularization due to in-stent restenosis or incomplete revascularization, with a retrospective study indicating recurrent angina rates of approximately 24.6% at 12 months, and female STEMI patients were more likely to experience recurrent angina (6). Furthermore, late recurrent angina after CABG has been documented in 18% of patients at 5 years, and during a median follow-up of 8.9 years, persistent angina was independently associated with higher rates of subsequent cardiac death (7). Early postoperative angina, particularly within the first month, is less well-characterized but is clinically critical, as it may signal procedural complications or suboptimal outcomes (8). Focusing on this one-month window is justified because early recurrence often reflects acute issues like incomplete revascularization or periprocedural injury, which differ from longer-term progression of disease; moreover, early prediction allows for timely interventions (e.g., medication adjustments or enhanced monitoring) to prevent escalation, reduce readmissions, and improve short-term quality of life, addressing a gap where most models target outcomes beyond 6–12 months (4, 8).

Traditional risk assessment tools, such as the SYNTAX score or Framingham risk score, rely on linear regression models and have limitations in capturing complex, non-linear interactions among patient variables, leading to suboptimal predictive accuracy for short-term events like one-month angina recurrence (9). Machine learning (ML) algorithms offer a promising alternative by leveraging large datasets to identify patterns and predict outcomes with greater precision. In cardiovascular medicine, ML has been increasingly applied for risk prediction, including major adverse cardiovascular events (MACE) following PCI (10, 11). For instance, studies have demonstrated that ML models outperform traditional scores in discriminating MACE and bleeding risks post-PCI, with area under the curve (AUC) values exceeding 0.80 in some validations (12). Metabolic signatures identified via ML have also been used to stratify patients at high risk for angina recurrence in remitted populations (13).

Several investigations have explored ML for predicting adverse outcomes after revascularization. One study developed early prediction models for MACE in acute myocardial infarction patients post-PCI, achieving robust performance through ensemble methods like random forests and XGBoost (14). Another validated an ML-based model for MACE following PCI, incorporating features such as Killip classification and Gensini score (15). ML has also been employed to predict composite cardiovascular events in stable angina patients, using risk score models that integrate pharmacological and clinical data (16). Artificial intelligence applications in coronary interventions have shown ML outperforming logistic regression for post-PCI outcomes like in-hospital mortality and heart failure (17).

Despite these advancements, gaps persist in the literature. Most existing ML models focus on long-term MACE or broad adverse events rather than specifically targeting short-term angina recurrence within one month post-revascularization. There is also a paucity of research integrating baseline clinical, laboratory, and psychological data for ultra-early predictions, which could enhance personalized medicine in the immediate postoperative phase. Addressing these gaps is crucial, as accurate early prediction could facilitate targeted interventions, such as intensified anti-ischemic therapy or closer monitoring, ultimately improving patient outcomes (16). Our working hypothesis was that a ML model incorporating readily available baseline variables (e.g., NYHA classification, cTnT, PT, depression severity, abdominal circumference, and DBP) would identify multifactorial predictors of one-month PRA, outperforming traditional tools by capturing non-linear interactions. We anticipated finding that cardiac (e.g., NYHA and cTnT), hemostatic (e.g., PT), psychological (e.g., depression), metabolic (e.g., abdominal circumference), and hemodynamic (e.g., DBP) factors would emerge as key predictors, as prior evidence suggests these domains influence early ischemic recurrence through mechanisms like ventricular dysfunction, thrombosis, stress-induced ischemia, obesity-related inflammation, and vascular instability. This approach builds on existing ML studies by shifting focus to short-term, actionable predictions using pre-procedural data, avoiding reliance on post-procedural complications that are not feasible for upfront risk stratification.

The objective of this study is to develop and validate a machine learning-based predictive model for angina pectoris recurrence one month after coronary revascularization. By harnessing advanced algorithms and comprehensive clinical datasets, we aim to provide a tool that enhances risk stratification and supports evidence-based clinical decision-making.

## Methods

### Research design

**Figure 1:**
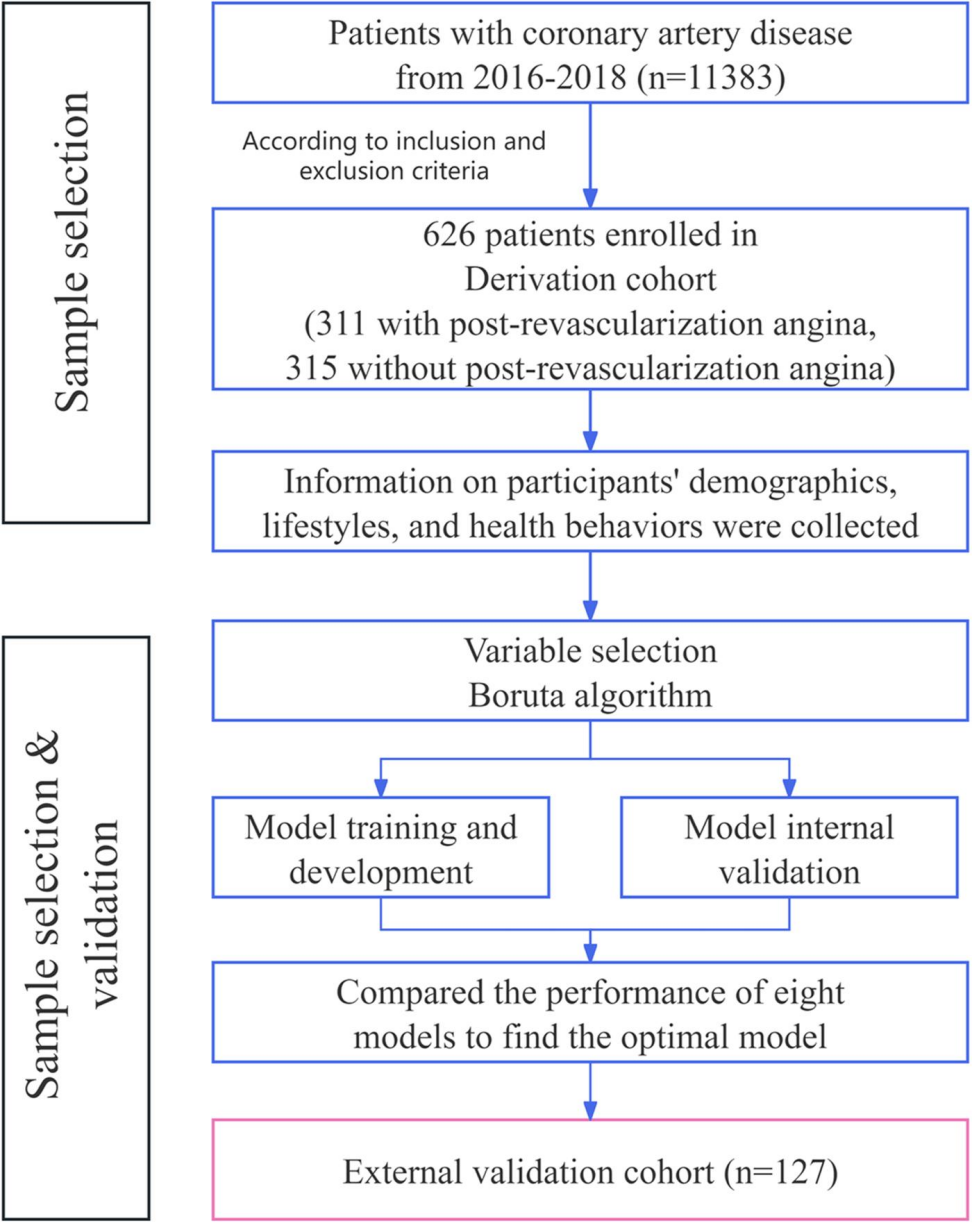
Flow diagram of the study population.

The research design for this study is depicted in Figure 1 and comprises of 4 steps: development, internal validation, external validation, and interpretation. Initially, a training cohort, constituting 70% of the derivation cohort, was used to develop predictive models. Subsequently, the remaining 30% of the derivation cohort was designed for internal validation, while an independent validation dataset was employed for external validation. The Shapley Additive explanations (SHAP) algorithm was utilized to elucidate the significance of features in the predictive model and to identify nonlinear relationships among risk predictors.

### Study subjects

The model was developed and internally validated in a population-based cohort from China, comprising over 11 000 participants aged 40–80 years at enrolment. Participants were recruited from 48 clinical research centers in 23 provinces in 5 regions (North China, Northeast China, Northwest China, Middle East China and South China) between 2016 and 2018. Supplementary Table S1 provides detailed information on clinical research centers, provinces, and regions. Cases were all outpatients or hospitalized patients with coronary artery disease. Among them, patients in both the PCI and CABG groups underwent four follow-up visits: within one week before surgery, within two weeks after surgery, one month after surgery, and six months after surgery. All participants underwent a baseline assessment, including a basic data collection form, physical examination, collection of blood samples and a series of questionnaires comprising the Seattle Angina Questionnaire (SAQ), the Patient Health Questionnaire (PHQ-9), and the Generalised Anxiety Disorder Questionnaire (GAD-7).

In the present study, we utilized data from all patients in the PCI group and the CABG group. The inclusion criteria were as follows: (1) Patients who underwent PCI or CABG and maintained follow-up appointments both one week prior to surgery and one month after surgery, to ensure complete baseline and short-term outcome data for assessing angina recurrence; (2) Patients aged 35 to 85 years, as coronary artery disease predominantly affects middle-aged and older adults, and this range minimizes heterogeneity from pediatric or extreme elderly populations where procedural risks and comorbidities differ significantly. Exclusion criteria included: (1) Patients with a history of infection, fever, trauma, burns, or surgery within one week prior to admission, or those with active tuberculosis or rheumatic/immune diseases, to avoid confounding effects from acute inflammatory states that could mimic or exacerbate angina symptoms; (2) Patients with severe arrhythmias (such as rapid atrial fibrillation, atrial flutter, paroxysmal ventricular tachycardia, etc.) accompanied by hemodynamic changes, as these could independently contribute to ischemic events and bias post-revascularization outcomes; (3) Patients with valvular heart disease (moderate or severe involvement in one or more valves) or primary cardiomyopathy, to exclude structural heart conditions that may cause persistent symptoms unrelated to coronary revascularization; (4) Patients with acute exacerbations of chronic obstructive pulmonary disease, pulmonary heart disease, or respiratory failure, to prevent respiratory comorbidities from influencing angina assessment or recovery; (5) Patients in the acute and subacute phases of cerebrovascular disease, as concurrent neurological events could confound symptom reporting and follow-up compliance; (6) Known renal insufficiency, with serum creatinine (Cr) levels exceeding 221 μmol/L in males and 177 μmol/L in females, to eliminate biases from impaired drug clearance and metabolic disturbances; (7) Known hepatic insufficiency, alanine aminotransferase (ALT) > 3 times the upper limit of normal, or with cirrhosis, for similar reasons related to metabolic and pharmacologic interactions; (8) Severe primary diseases such as combined hematopoietic system disorders or malignant tumors, to avoid complications from systemic illnesses that could affect cardiovascular recovery; (9) Organ transplant patients, due to immunosuppressive therapy and altered immune responses that may impact healing and ischemia; (10) Patients with severe mental disorders (perceptual disturbances, thought disorders), to ensure reliable self-reported angina symptoms via questionnaires; (11) Pregnant women and breastfeeding women, as pregnancy and lactation alter cardiovascular hemodynamics, increase procedural risks (e.g., radiation exposure in PCI), and may contraindicate certain medications used in post-revascularization care.

The definition of post-revascularization angina is a Seattle Angina Questionnaire-Angina Frequency (SAQ-AF) score below 100 at the 1-month follow-up. The scale in question is a tool designed to assess patient status by quantifying the frequency and severity of angina symptoms. Specifically, this subscale gauges the frequency of angina attacks experienced by participants over the preceding four weeks, with scores ranging from 0 (daily episodes) to 100 (no episodes). A score below 100 indicates that the subject experienced at least one angina episode during the follow-up period. This threshold is clinically meaningful as it aligns with validated SAQ cutoffs where scores <100 correlate with impaired quality of life and increased healthcare utilization, though it may include minor episodes; this broadens the model's utility for early screening to capture at-risk patients for preventive interventions, potentially over-identifying cases but prioritizing sensitivity in high-stakes settings (4).

This study used patient data from 38 clinical research centers in 23 provinces of China from 2016 to 2018. The derivation cohort included patients who underwent PCI or CABG at 28 clinical research centers across 16 provinces. A total of 1,003 individuals meeting these criteria were screened for participation. Based on the exclusion criteria, 324 patients had conditions that could potentially affect the final outcome, while 53 patients had incomplete or missing data. Consequently, a total of 626 eligible patients were selected for the derivation cohort. Among these, 311 patients met the diagnostic criteria for post-revascularization angina and constituted the PRA group, while the remaining 315 patients formed the non-PRA group. Table 1 presents an overview of the clinical characteristics of the 626 patients included in the derivation cohort.

**Table 1:** Baseline characteristics of All participants.

Characteristic	Overall	Derivation cohort	External validation cohort	*p*-value
Participants, n	753	626	127	
PRA, *n*				0.617
No	382 (50.73%)	315 (50.32%)	67 (52.76%)	
Yes	371 (49.27%)	311 (49.68%)	60 (47.24%)	
Age, median (IQR), y	63 (56–70)	63 (55–70)	64 (57–71)	0.542
Gender, *n* (%)				1.000
Male	540 (71.71)	449 (71.73)	91 (71.65)	
Female	213 (28.29)	177 (28.27)	36 (28.35)	
Region, *n* (%)				0.205
North CN	36 (4.78)	28 (4.47)	8 (6.30)	
Northeast CN	16 (2.12)	13 (2.08)	3 (2.36)	
Middle East CN	241 (32.01)	208 (33.23)	33 (25.98)	
Northwest CN	108 (14.34)	83 (13.26)	25 (19.69)	
South CN	352 (46.75)	294 (46.96)	58 (45.67)	
Marital status, *n* (%)				1.000
Married/cohabiting	711 (94.42)	591 (94.41%)	120 (94.49%)	
Widowed/never married/divorced	42 (5.58)	35 (5.59%)	7 (5.51%)	
Education level, *n* (%)				0.192
Middle school and below	451 (59.89)	382 (61.02%)	69 (54.33%)	
High school and above	302 (40.11)	244 (38.98%)	58 (45.67%)	
NYHA classification, *n* (%)				0.945
Class I	326 (43.29%)	272 (43.45%)	54 (42.52%)	
Class II	317 (42.10%)	264 (42.17%)	53 (41.73%)	
Class III	101 (13.41%)	82 (13.10%)	19 (14.96%)	
Class IV	9 (1.20%)	8 (1.28%)	1 (0.79%)	
MPA, d/wk	4 (2–7)	4 (2–7)	4 (2–7)	0.524
Drinking, *n* (%)				0.845
No	627 (83.27)	520 (83.07%)	107 (84.25%)	
Yes	126 (16.73)	106 (16.93%)	20 (15.75%)	
Smoking, *n* (%)				0.582
No	563 (74.77%)	471 (75.24%)	92 (72.44%)	
Yes	190 (25.23%)	155 (24.76%)	35 (27.56%)	
Abdominal circumference, cm	90.00 (80.00–99.00)	90.00 (81.00–99.00)	88.00 (79.50–98.00)	0.175
Waist hip ratio	0.93 (0.82–0.98)	0.92 (0.80–0.98)	0.93 (0.84–1.00)	0.182
Pulse rate	74.00 (68.00–81.00)	74.00 (68.00–82.00)	75.00 (68.00–80.00)	0.746
SBP	131.00 (120.00–145.00)	130.00 (120.00–145.00)	132.00 (120.00–144.00)	0.799
DBP	80.00 (70.00–90.00)	80.00 (71.00–90.00)	80.00 (70.00–85.00)	0.109
Depression, *n* (%)				0.089
None—minimal	561 (74.50)	472 (75.40%)	89 (70.08%)	
Mild	145 (19.26)	121 (19.33%)	24 (18.90%)	
Moderate	37 (4.91)	27 (4.31%)	10 (7.87%)	
Moderately severe	8 (1.06)	5 (0.80%)	3 (2.36%)	
Severe	2 (0.27)	1 (0.16%)	1 (0.79%)	
Anxiety disorder, *n* (%)				0.062
None—minimal	630 (83.67)	526 (84.03%)	104 (81.89%)	
Mild	92 (12.22)	79 (12.62%)	13 (10.24%)	
Moderate	25 (3.32)	18 (2.88%)	7 (5.51%)	
Severe	6 (0.80)	3 (0.48%)	3 (2.36%)	
BMI, Kg/m^2^	24.36 (22.27–26.89)	24.42 (22.20–26.89)	24.21 (22.76–26.96)	0.745
FBG, mmol/L	5.55 (4.87–6.96)	5.51 (4.82–6.89)	5.91 (5.01–7.64)	0.079
HDL, mmol/L	1.06 (0.89–1.29)	1.06 (0.89–1.29)	1.06 (0.90–1.27)	0.889
LDL, mmol/L	2.63 (1.98–3.26)	2.65 (2.01–3.28)	2.49 (1.86–3.17)	0.105
CRP, mg/L	3.13 (1.00–8.50)	3.48 (1.00–8.50)	2.90 (0.86–8.52)	0.513
TC, mmol/L	4.31 (3.52–4.99)	4.28 (3.57–5.00)	4.33 (3.40–4.97)	0.576
TG, mmol/L	1.39 (1.03–1.92)	1.40 (1.04–1.92)	1.37 (1.01–1.96)	0.857
UA, μmol/L	330.00 (270.00–388.00)	328.00 (270.00–386.00)	334.00 (274.00–403.00)	0.531
ApoA-I/ApoB	1.30 (1.01–1.80)	1.29 (1.01–1.77)	1.47 (1.01–1.92)	0.293
PT, s	12.00 (11.10–12.90)	11.95 (11.10–12.90)	12.10 (10.80–13.10)	0.479
TT, s	16.20 (13.80–17.70)	16.10 (13.80–17.70)	16.40 (14.40–17.85)	0.555
FIB, g/L	3.16 (2.63–3.79)	3.17 (2.64–3.79)	3.11 (2.61–3.77)	0.371
APTT, s	31.60 (27.60–36.00)	31.45 (27.40–35.80)	32.40 (28.30–36.20)	0.298
LDH, U/L	202.92 (158.00–395.00)	205.50 (159.00–399.95)	195.00 (157.11–366.85)	0.575
CKMB, U/L	13.00 (8.00–23.29)	13.00 (8.00–23.29)	11.00 (6.46–22.70)	0.106
cTnT, μg/L	0.01 (0.01–0.08)	0.01 (0.01–0.09)	0.02 (0.01–0.07)	0.145
LVEF	0.60 (0.56–0.66)	0.60 (0.56–0.66)	0.61 (0.56–0.66)	0.521

Data are expressed as *n* (%) or median (interauartile range), *P*-values are obtained from Mann–Whitney *U*-test or chi-square test.

PRA, post-revascularization angina; NYHA classification, The New York Heart Association classification; MPA, moderate physical activity; SBP, systolic blood pressure; DBP, diastolic blood pressure; BMI, body mass index; FBG, fasting blood glucose; HDL, high-density lipoprotein cholesterol; LDL, low-density lipoprotein cholesterol; CRP, C-reactive protein; TC, total cholesterol; TG, triglyceride; UA, uric acid; ApoA-I, Apolipoprotein A-I; ApoB, Apolipoprotein B; PT, prothrombin time; TT, thrombin time; FIB, fibrinogen; APTT, activated partial thromboplastin time; LDH, lactatedehydrogenase; CKMB, creatine kinase-MB; cTnT, cardiac troponin T; LVEF, Left Ventricular Ejection Fraction.

The external validation cohort consisted of 127 patients, recruited from another 10 clinical research centers across 10 provinces in China between 2016 and 2018. Among these, 60 patients had scores that met diagnostic criteria for PRA, forming the PRA group. The remaining 67 patients constitute non-PRA group. Table 1 presents an overview of the clinical characteristics of the 127 patients included in the external validation cohort. The external cohort size was determined based on power calculations assuming an expected AUC of 0.85 (from internal validation), a PRA prevalence of 50% (as observed in derivation), and a desired power of 80% with alpha=0.05, yielding a minimum of 100 patients for detecting model performance degradation; the 127 patients exceeded this, supporting adequate generalizability across different centers, though the disparity with the derivation cohort reflects real-world data availability from independent sites.

### Data collection

Information on participants' demographics, lifestyles, and health behaviors were collected by uniformly trained enumerators using a group-developed information collection form. Demographic data includes sex, age, body mass index (BMI), waist, abdominal, and hip circumferences, religion, job, place of residence, moderate physical activity (MPA), smoking status (current vs. never/ former), drinking status (current vs. never/ former), The New York Heart Association (NYHA) classification, systolic blood pressure (SBP), diastolic blood pressure (DBP). Laboratory tests mainly include C-reactive protein (CRP, mg/L), HDL cholesterol (HDL, mmol/L), LDL cholesterol (LDL, mmol/L), fasting blood glucose(FBG, mmol/L), ApoA-Ⅰ (g/L), ApoB (g/L), Triglyceride (TG, mmol/L), Total Cholesterol (TC, mmol/L), homocysteine (HCY, pg/mL), uric acid (UA, μmol/L), prothrombin time (PT, s); thrombin time (TT, s); fibrinogen (FIB, g/L); activated partial thromboplastin time (APTT, s); lactatedehydrogenase (LDH, U/L); creatine kinase-MB (CKMB, U/L); cardiac troponin T (cTnT, μg/L); left ventricular ejection fraction (LVEF). The Chinese version of Patient Health Questionnaire (PHQ-9), and the Generalised Anxiety Disorder Questionnaire (GAD-7) from the website of the National Mental Health Database was used to assess depression and anxiety symptoms. The higher the score, the more severe the symptoms of depression and anxiety will be. The Chinese version of the Seattle Angina Questionnaire (SAQ) from the website of the National Center For Cardiovascular Disease was used to assess the quality of survival in patients with stable angina pectoris. The SAQ angina frequency scale range from 0 to 100 points, with higher scores indicating less angina.

### Statistical analyses

Baseline data analysis of patients began with normality tests on the quantitative data. This study used the Kolmogorov–Smirnov test to assess the normality of data distribution for continuous variables. The data on normal distribution was represented by the mean and standard deviation and was compared using the independent samples t-test. Data that did not follow a normal distribution were presented using the median and interquartile range and were compared using the Mann–Whitney U test. Descriptive statistics was conducted with frequencies for categorical variables presented as percentages (%) and using the chi-square or Fisher exact tests for comparison. All continuous variables underwent preprocessing prior to model training. Z-score standardization was applied to laboratory indicators (e.g., CRP, LDH) and anthropometric measures (BMI, waist circumference) to mitigate scale differences between features. Variables with >15% missingness were excluded; remaining missing values were imputed using multiple imputation by chained equations with predictive mean matching (5 iterations). Missingness rates ranged from 2%–12% across retained variables (e.g., 8% for cTnT, 5% for depression severity), with patterns appearing missing at random based on Little's test (*p* > 0.05); sensitivity analyses using complete-case data yielded similar model performance (AUC change <0.02), suggesting minimal bias in this retrospective multi-center context, though non-random patterns in smaller sites could subtly affect reliability. The Boruta algorithm was used to finalize the variables for inclusion, thereby eliminating any redundant features. A two-tailed *P*-value of less than 0.05 was deemed statistically significant.

### Variable selection

The Boruta algorithm was a wrapper method for feature selection built around the Random Forest Classifier algorithm. During the model construction, Boruta created a copy of the original dataset features as Shadow Features and compared the Z-score between the actual features and shadow features calculated via Random Forest Classifier in each iteration. If the Z-score of an actual feature was higher than the maximum Z-score of shadow features, this feature was considered pivotal and kept; otherwise, it was dropped. While Boruta's rigorous comparison reduces overfitting by excluding redundant or noisy variables, it may omit factors like LDL, smoking status, and LVEF (which showed group differences in Table 1) if they do not independently contribute beyond the selected features in the RF context; for example, these could be correlated with retained variables (e.g., abdominal circumference for metabolic risk, NYHA for functional status), as Boruta prioritizes unique importance.

### Model derivation and validation

The machine learning algorithm models were developed using R software package (version 4.2.1). In this study, eight machine learning algorithms—Decision Tree (DT), Logistic Regression (Logistic), K-nearest Neighbor (KNN), Naive Bayesian (NB), light gradient boosting machine (LightGBM), Extreme Gradient Boosting (Xgboost), Support Vector Machine (SVM) and Random Forest (RF)— were utilized to construct the post-revascularization angina prediction model. The models were constructed as follows: The DT model using the rpart package; the Logistic model using the glm package; the KNN model using the kknn package; the NB model using the klaR package; the LightGBM model using the lightgbm package; the Xgboost model using the xgboost package; the SVM model using the e1071 package; and the RF model using the randomForest package. To substantiate claims of ML superiority over traditional tools, we included a baseline comparison with logistic regression (as a proxy for linear models like SYNTAX/Framingham scores) using the same features, evaluating it alongside the ML models.

Each classification algorithm underwent hyperparameter tuning through 5-fold cross-validation internally. The loss function was being minimized through cross-validation to find the optimal hyperparameters. We evaluated the prediction power of each machine learning classifier using the optimal hyperparameters on the internal and external validation sets, and the performance of the models was compared using metrics such as the area under the curve, accuracy, sensitivity, specificity, and F1 score. Decision curve analysis (DCA) was performed on the testing set to evaluate the value and relative superiority of each model in the applied scenario. The Shapley Additive explanations (SHAP) algorithm was utilized to elucidate the significance of features in the predictive model and to identify nonlinear relationships among risk predictors.

### Ethics statement

This study was approved by the institute of Basic Theory for Chinese Medicine China Academy of Chinese Medical Sciences Medical Ethics Committee (No.2016EC_KY_001), and written informed consent was obtained from all participants.

### Role of the funding source

The funding source had no role in the study design, data collection, analysis, interpretation of data, the writing of the report, or in the decision to submit the paper for publication. All authors had full access to all data in the study and had final responsibility for the decision to submit for publication.

## Results

### Patient characteristics

This study conducted an initial comparison between the PRA group (374 individuals) and the Non-PRA group (379 individuals). The comparison focused on their baseline characteristics including age at first admission, sex, marital status, education level, The New York Heart Association (NYHA) classification, anxiety and depression level, BMI, waist hip ratio, life style, left ventricular ejection fraction (LVEF) and the baseline levels of 15 serum indicators (Table 1). The median age was 63 years, with 71.71% being male. Compared with those in the Non-PRA group, people in the PRA group were more likely to reside in middle east China. They exhibited a higher prevalence of NYHA Class II and III, engaged in less weekly physical activity, were more likely to be current smokers, and had larger abdominal circumferences. Additionally, they had higher systolic blood pressure, experienced depressive states, and had elevated CRP and FIB levels. They also had lower PT, LDH, and LVEF values.

### Variable selection

**Figure 2:**
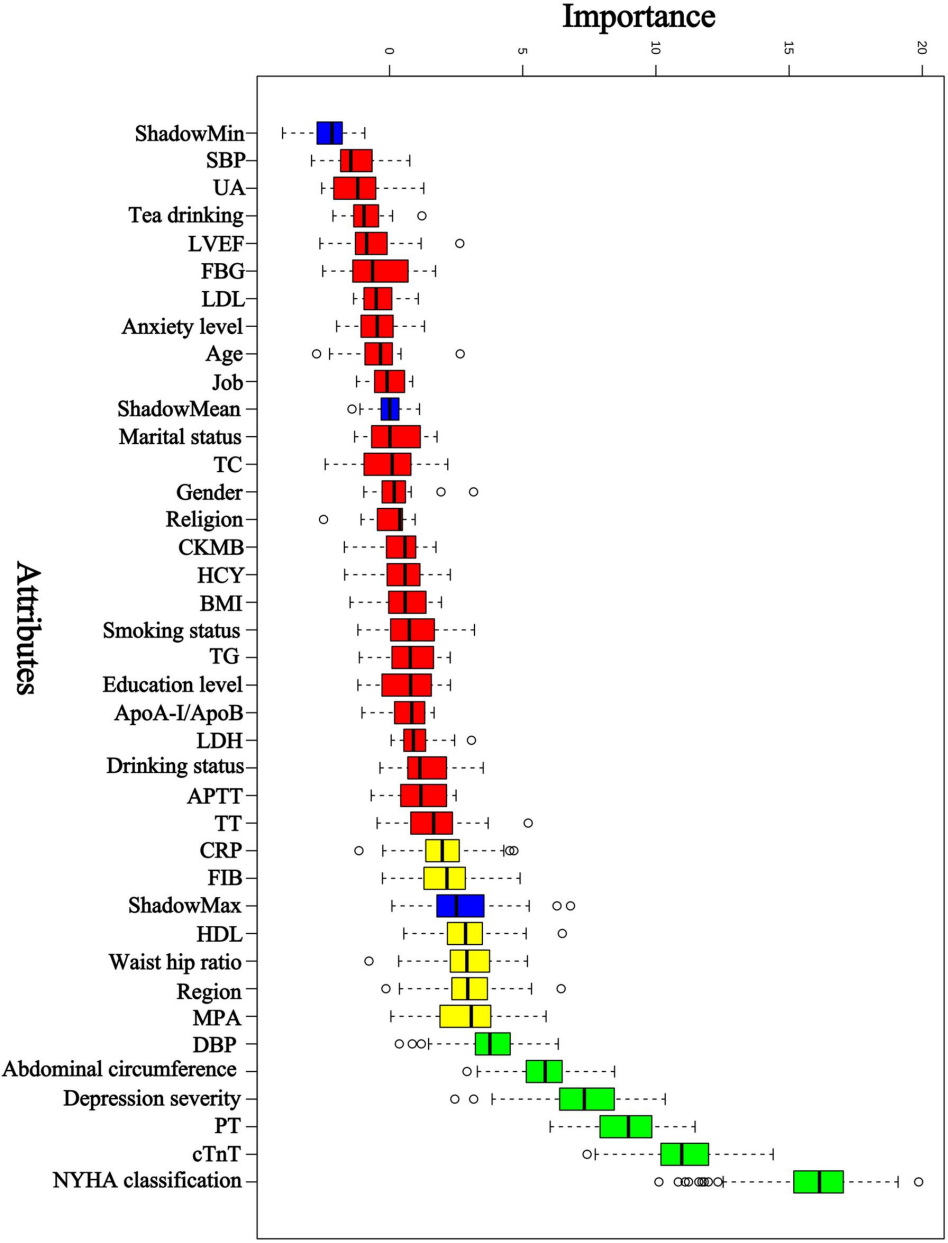
Predictor selection using Boruta. NYHA classification, The New York Heart Association classification; cTnT, cardiac troponin T; PT, prothrombin time; DBP, diastolic blood pressure; MPA indicates moderate physical activity; HDL, high-density lipoprotein cholesterol; FIB, fibrinogen; CRP, C-reactive protein; TT, thrombin time; APTT, activated partial thromboplastin time; LDH, lactatedehydrogenase; ApoA-I, Apolipoprotein A-I; ApoB, Apolipoprotein B; TG, triglyceride; BMI, body mass index; HCY, homocysteine; CKMB, creatine kinase-MB; TC, total cholesterol; LDL, low-density lipoprotein cholesterol; FBG, fasting blood glucose; LVEF, left ventricular ejection fraction; UA, uric acid; SBP, systolic blood pressure.

Based on previous clinical work, theoretical analysis, and expert consultation, we initially identified 36 predictors: age, gender, region, marital status, education, religion, job, MPA, smoking status, drinking status, tea drinking, anxiety level, depression severity, NYHA classification, abdominal circumference, waist hip ratio, pulse rate, SBP, DBP, BMI, CRP, HDL, LDL, FBG, ApoA-I/ApoB, TG, TC, UA, PT, TT, FIB, APTT, LDH, CKMB, cTnT, LVEF. Boruta was then used for further feature selection. Boruta selected 6 predictors: NYHA classification, cTnT, PT, depression severity, abdominal circumference, and DBP. The results are shown in Figure 2. The selected 6 predictors were included as the risk factors to develop ML model (PRA or non-PRA).

### Development and evaluation of the PRA diagnostic model

**Figure 3:**
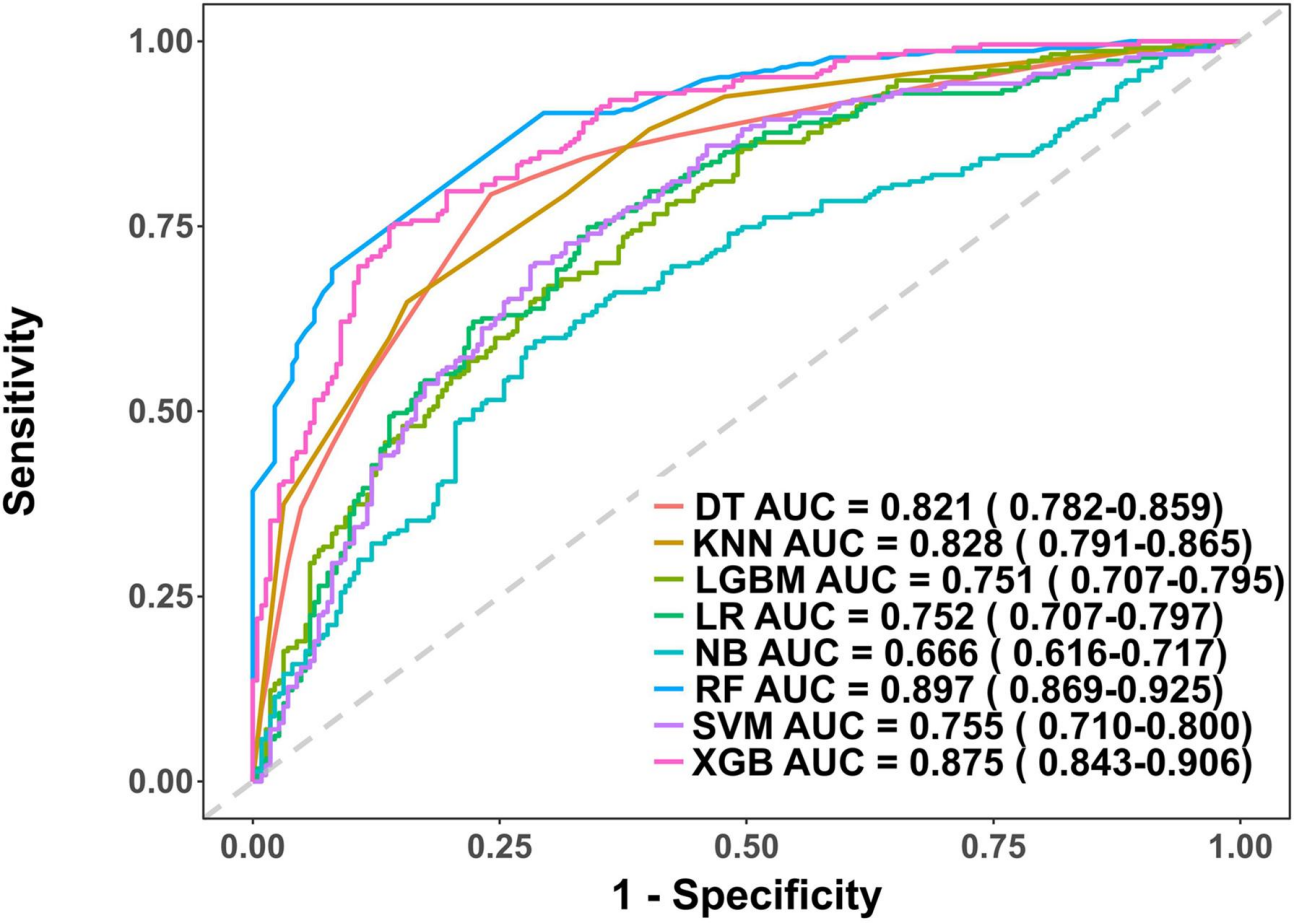
Receiver operating characteristic (ROC) curves for each model in the internal validation cohort. DT, Decision tree model; KNN, K-Nearest Neighbor model; LGBM, Light Gradient Boosting Machine model; LR, Logistic regression model; NB, Naive Bayes model; RF, Random forest model; XGB, Extreme gradient boosting model; SVM, Support Vector Machine.

**Figure 4:**
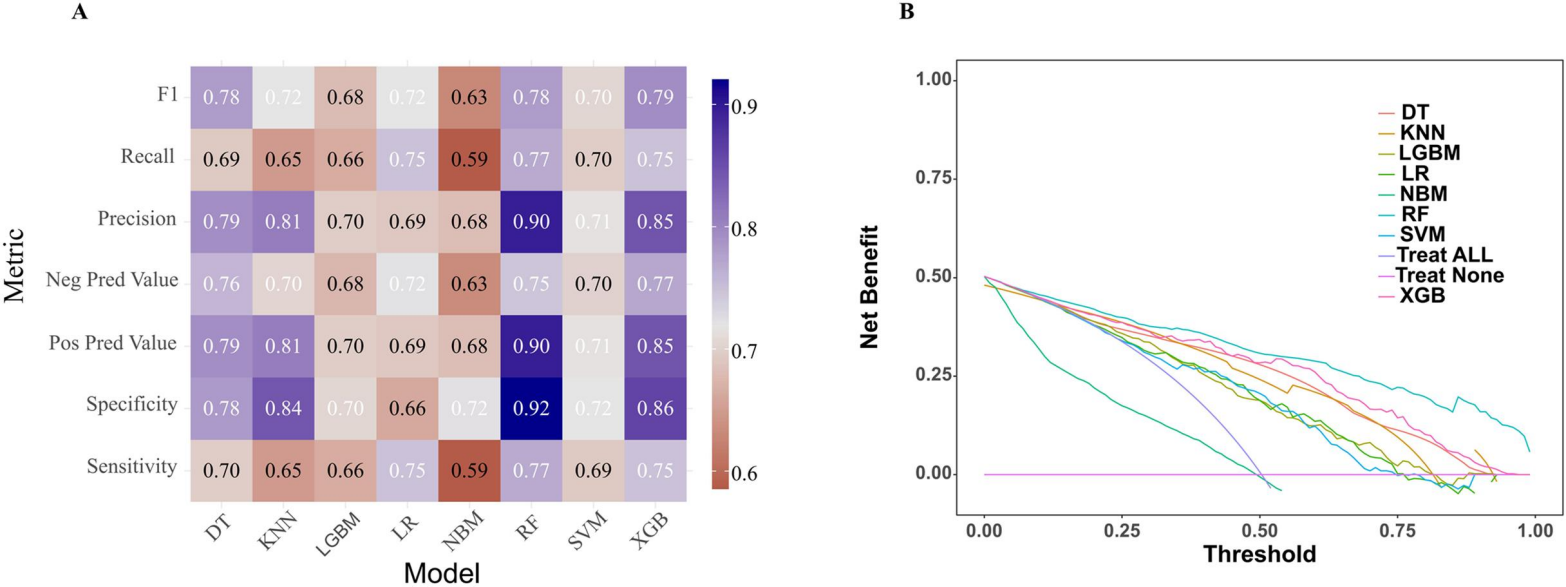
Comparative performance analysis of eight models in the internal validation cohort. **(A)** Multi-metric performance summary of each model for PRA in the internal validation cohort. **(B)** Decision curve analysis (DCA) curves for each model in the internal validation cohort. PRA, post-revascularization angina.

In the model training, a positive class represented the presence of PRA, while a negative class represented the absence of PRA. Following variable selection, the input data to train the model consisted of serum levels for 2 indicators closely related to the mechanism of angina pectoris, in addition to NYHA classification, depression severity, abdominal circumference, and DBP of patients at admission. Utilizing these features, we developed eight different machine learning models, including DT, Logistic, KNN, NB, LGBM, Xgboost, SVM and RF. The findings of this study demonstrate that the RF model displayed a significantly higher AUC value in comparison to other machine learning algorithms in the internal validation cohorts (Figure 3). Further examination of the data in the internal validation cohort revealed that the RF model exhibited an accuracy of 0.88, a sensitivity of 0.77, a specificity of 0.92, an F1 score of 0.78, and an AUC value of 0.90 (Figure 3, Figure 4A and Table 2). These outcomes strongly suggest that the RF model surpassed the other seven models in terms of various performance parameters, including the baseline logistic regression (AUC 0.74), substantiating ML's ability to capture non-linear interactions better than traditional linear approaches. Decision Curve Analysis (DCA) is a straightforward method to evaluate the clinical utility of disease diagnostic models. The DCA curve depicted in Figure 4B further demonstrated that the RF model had the highest clinical utility.

**Table 2:** Diagnostic performance of each model for PRA in internal validation cohort.

Model	Sensitivity	Specificity	Pos Pred Value	Neg Pred Value	Precision	Recall	F1	Accuracy
LR	0.75	0.66	0.69	0.72	0.69	0.75	0.72	0.64
DT	0.70	0.78	0.79	0.76	0.79	0.69	0.78	0.86
RF	0.77	0.92	0.90	0.75	0.90	0.77	0.78	0.88
XGB	0.75	0.86	0.85	0.77	0.85	0.75	0.79	0.84
SVM	0.69	0.72	0.71	0.70	0.71	0.70	0.70	0.66
KNN	0.65	0.84	0.81	0.70	0.81	0.65	0.72	0.72
LGBM	0.66	0.70	0.70	0.68	0.70	0.66	0.68	0.68
NB	0.59	0.72	0.68	0.63	0.68	0.59	0.63	0.62

LR, Logistic regression model; DT, Decision tree model; RF, Random forest model; XGB, Extreme gradient boosting model; SVM, Support vector machine model; KNN, K-Nearest Neighbor model; LGBM, Light Gradient Boosting Machine model; NB, Naive Bayes model.

### External validation of the PRA diagnostic model

**Figure 5:**
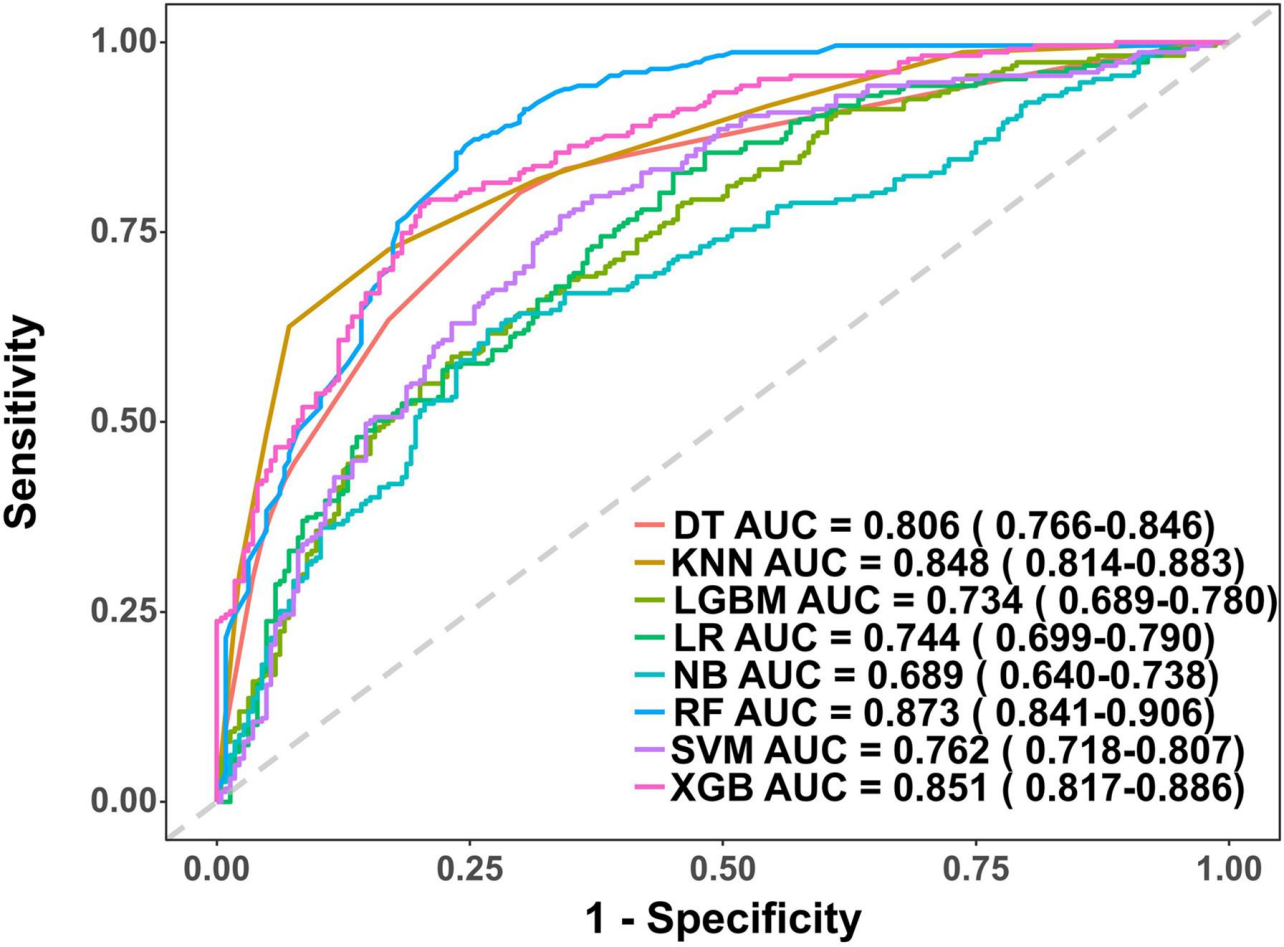
Receiver operating characteristic (ROC) curves for each model in the external validation cohort. DT, Decision tree model; KNN, K-Nearest Neighbor model; LGBM, Light Gradient Boosting Machine model; LR, Logistic regression model; NB, Naive Bayes model; RF, Random forest model; XGB, Extreme gradient boosting model; SVM, Support Vector Machine.

**Figure 6:**
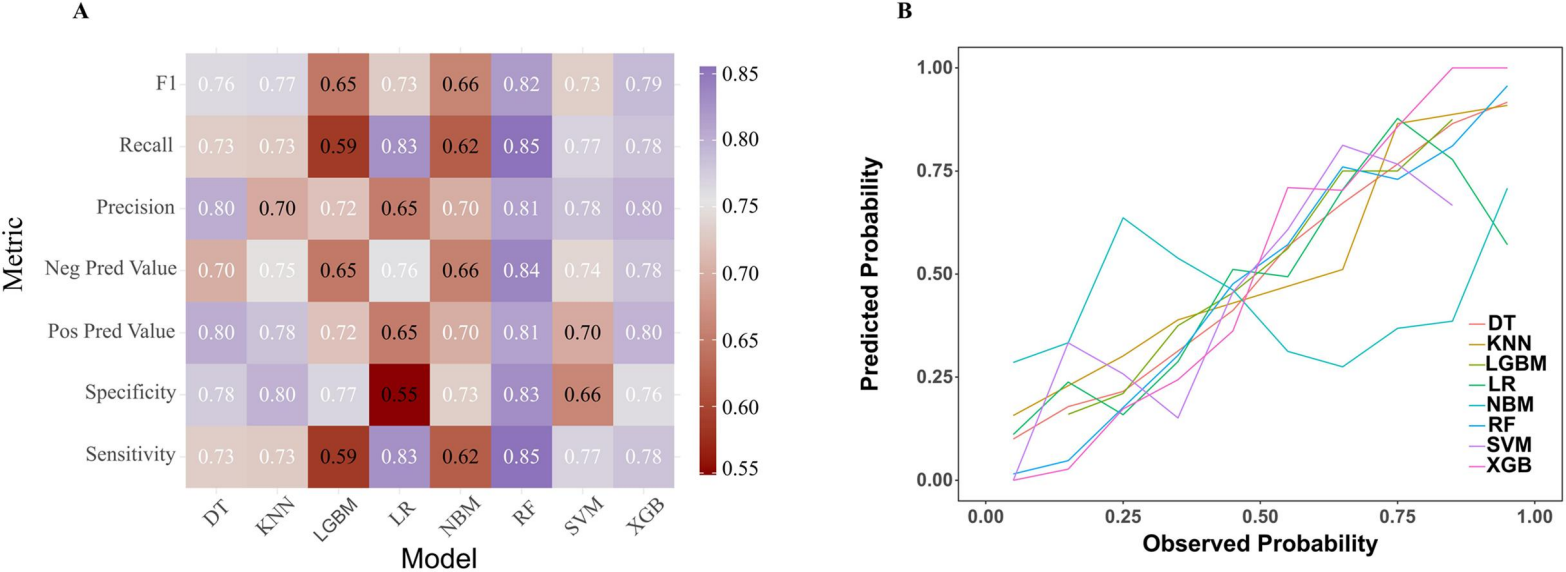
Comparative performance analysis of eight models in the external validation cohort. **(A)** Multi-metric performance summary of each model for PRA in the internal validation cohort. **(B)** Calibration characteristics analysis for each model in the external validation cohort. PRA, post-revascularization angina.

This study involved conducting an external validation and performance comparison of seven machine learning models using a cohort of 127 angina patients from another 10 clinical research centers across 10 provinces in China. In terms of predictive performance, the results obtained from the external validation cohort indicated that the RF model achieved an AUC of 0.87, followed by the Xgboost model with an AUC of 0.85, the KNN model with an AUC of 0.85, the DT model with an AUC of 0.81, the SVM model with an AUC of 0.76, the Logistic model with an AUC of 0.74, the LGBM model with an AUC of 0.73, and the NB model with an AUC of 0.69, as depicted in Figure 5. Furthermore, provides an overview of the accuracy, sensitivity, specificity, and F1 Score of the eight models in the external validation cohort was provided (Table 3, Figure 6A). Data visualization was performed to assess the predictive performance of the eight models on the external validation cohort. The calibration curve (Figure 6B) demonstrated a satisfactory alignment between the predicted risk of the RF model and the observed risk. To summarize, the outcomes of the external confirm that the RF model demonstrates higher performance in the early prediction of PRA (Figure 6B).

**Table 3:** Diagnostic performance of each model for PRA in external validation cohort.

Model	Sensitivity	Specificity	Pos Pred Value	Neg Pred Value	Precision	Recall	F1	Accuracy
LR	0.83	0.55	0.65	0.76	0.65	0.83	0.73	0.65
DT	0.73	0.78	0.80	0.70	0.80	0.73	0.76	0.68
RF	0.85	0.83	0.81	0.84	0.81	0.85	0.82	0.88
XGB	0.78	0.76	0.80	0.78	0.80	0.78	0.79	0.80
SVM	0.77	0.66	0.70	0.74	0.78	0.77	0.73	0.72
KNN	0.73	0.80	0.78	0.75	0.70	0.73	0.77	0.75
LGBM	0.59	0.77	0.72	0.65	0.72	0.59	0.65	0.63
NB	0.62	0.73	0.70	0.66	0.70	0.62	0.66	0.59

LR, Logistic regression model; DT, Decision tree model; RF, Random forest model; XGB, Extreme gradient boosting model; SVM, Support vector machine; KNN, K-Nearest Neighbor model; LGBM, Light Gradient Boosting Machine model; NB, Naive Bayes model.

### Model interpretation

**Figure 7:**
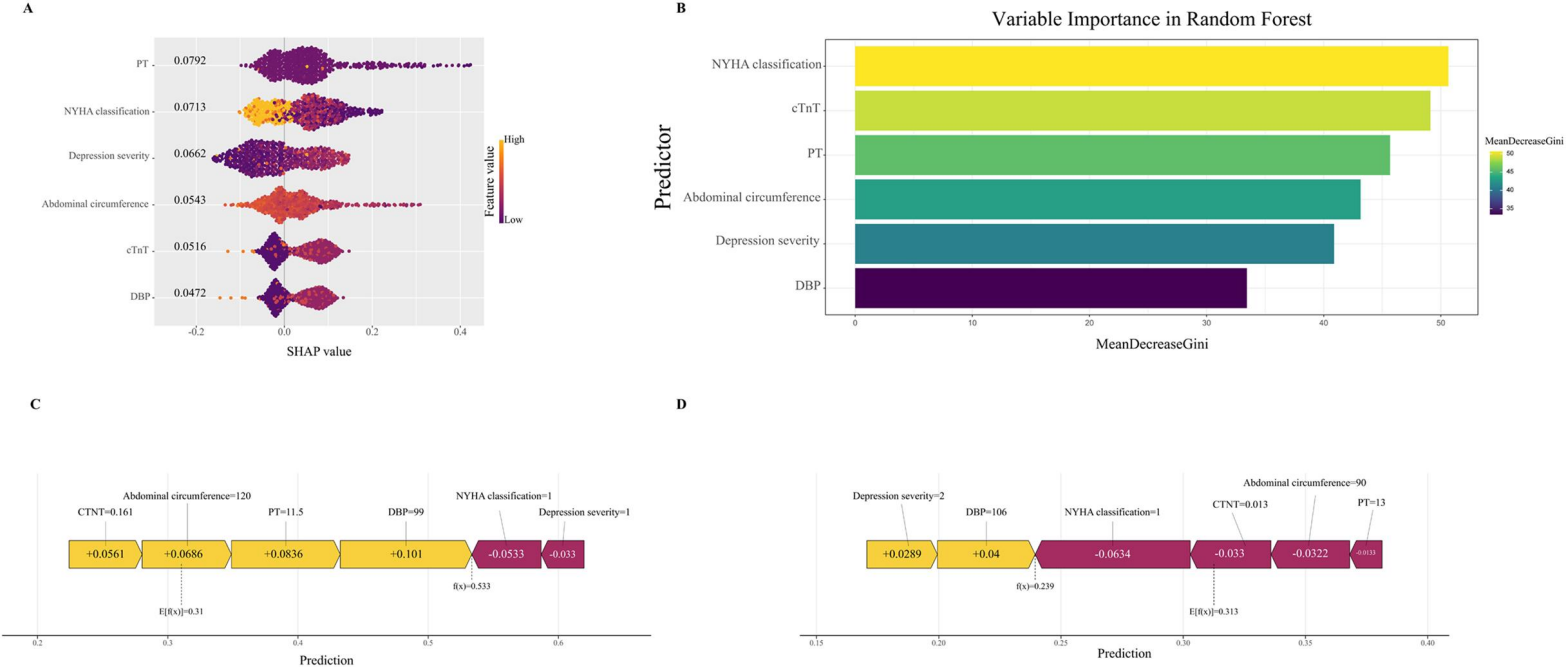
Interpretable machine learning analysis of risk factors. **(A)** All samples and features are illustrated, with each row representing a feature and x-axis representing the SHAP value. The purple dots represent higher feature values, while the yellow dots represent lower feature values. **(B)** Ranking of variable importance based on the average value. **(C)** SHAP predictions for PRA samples. **(D)** SHAP predictions for non-PRA samples. Yellow arrows indicate a higher risk of PRA, while purple arrows indicate a lower risk of PRA. The length of the arrows helps visualize the degree of impact of the prediction, whereby the longer the arrow, the more significant the effect. SHAP, The Shapley Additive explanations; PRA, post-revascularization angina.

To ensure a comprehensive understanding of the selected variables, we employed the SHAP algorithm to highlight their predictive importance in the optimal RF model for heat-sensitive angina. Figure 7A visually demonstrates the 6 key features of the RF model, including NYHA classification, cTnT, PT, abdominal circumference, depression severity, and DBP. The influence of each feature is illustrated by uniquely colored dots: The purple dots represent higher feature values, while the yellow dots represent lower feature values. Figure 7B depicts the hierarchical organization of these 6 risk factors, underlining their significance in the model. The *x*-axis, representing SHAP values, indicates the importance of each factor. The strong link between the 2 serum indicators and the PRA suggests their value as dependable indicators for the clinically detecting this type of disease progression. Additionally, we provide two typical examples, one predicting PRA (Figure 7C) and the other predicting non-PRA (Figure 7D), to demonstrate the model's interpretability.

## Discussion

In this retrospective cohort study, we developed and validated a machine learning-based predictive model for one-month post-revascularization angina (PRA) using baseline clinical data from 753 Chinese patients who had undergone PCI or CABG. The Boruta algorithm identified six key features—NYHA classification, cTnT, PT, depression severity, abdominal circumference, and DBP—which were integrated into eight ML algorithms, with the random forest (RF) model demonstrating superior performance (AUC 0.90 in internal validation and 0.87 in external validation). SHAP analysis further elucidated feature contributions, highlighting their positive or negative influences on PRA predictions.

Our findings on the predictive features align with and extend prior research on post-revascularization outcomes. For instance, NYHA classification as a top predictor is consistent with studies showing that higher NYHA classes (III/IV) are associated with increased angina recurrence and repeat interventions after CABG or PCI, likely due to underlying ventricular dysfunction (5, 18, 19). Similarly, elevated cTnT levels, indicative of myocardial injury, have been linked to refractory angina and recurrent ischemia post-PCI in previous work, where even minor elevations correlated with adverse events (20–22). This supports our model's emphasis on cTnT as a strong early marker, though our focus on baseline (pre-procedural) levels differentiates it from studies relying on post-procedural elevations. PT's role in our model echoes evidence of coagulation abnormalities contributing to thrombotic risks and worse outcomes after revascularization, as prolonged PT has been associated with mortality in ACS patients undergoing PCI (23). Compared to traditional predictors like age or smoking in earlier studies, our inclusion of PT adds novelty by highlighting hemostatic factors in short-term PRA, which are less emphasized in long-term MACE models (10). Depression severity's prominence aligns with research demonstrating that pre-existing depression and anxiety exacerbate short-term angina recurrence and major cardiac events post-PCI, underscoring the psychosomatic factors in recovery—potentially through mechanisms like increased inflammation, sympathetic activation, or stress-induced ischemia (24–26). However, depression may also serve as a proxy for unmeasured factors (e.g., poor medication adherence or sedentary lifestyle), which could reduce model interpretability if not accounted for; SHAP helps mitigate this by quantifying its independent contribution, but future models should explore mediation analyses. Abdominal circumference, as a proxy for central obesity, corroborates associations between waist circumference, obesity, and increased cardiovascular events or mortality after PCI in ACS cohorts, though its variable impact on revascularization outcomes in prior analyses suggests context-specific relevance (27–29). Finally, lower DBP's association with PRA persistence is in line with studies linking pre-procedural DBP to long-term mortality and recurrent events post-PCI, extending this to short-term angina by implying vascular instability as a mechanism (30, 31). Collectively, these features underscore the multifactorial etiology of PRA, integrating cardiac, hemostatic, psychological, and metabolic dimensions—contrasting with earlier models that often prioritized procedural or demographic factors alone (10, 32). Our RF model's superior AUC (0.87–0.90) outperforms many reported ML models for MACE post-PCI (AUC ∼0.80), likely due to its focus on short-term outcomes and ensemble methods like RF, which handle non-linear interactions better than logistic regression in comparable studies (12, 14, 15, 17). The slight AUC decline from 0.90 (internal) to 0.87 (external) may stem from institutional variations in data collection or patient demographics across provinces, suggesting modest limitations in geographic generalizability despite the multi-center design.

The implications of these new findings are significant for clinical practice and research. By identifying baseline predictors like depression and PT—often overlooked in routine assessments—our model enables early, personalized risk stratification, potentially guiding interventions such as psychological support or anticoagulation optimization to mitigate PRA. This addresses a gap in prior work, where models typically target long-term MACE rather than one-month recurrence, offering a tool for immediate post-discharge monitoring and reduced readmissions (16). Furthermore, SHAP's interpretability enhances model transparency, a common critique of ML in medicine, allowing clinicians to understand feature impacts and build trust in AI-driven decisions. The model's sensitivity (0.77) vs. specificity (0.92) trade-off prioritizes avoiding false positives in resource-limited settings, but for screening high-risk patients, higher sensitivity might be desirable to minimize missed cases; this could be adjusted via threshold tuning in implementation, balancing early intervention benefits against over-treatment risks.

Strengths include the use of Boruta for unbiased feature selection and multi-cohort validation, enhancing generalizability. Limitations encompass the retrospective design, potential selection bias, and the model was developed using data primarily from Chinese patients (2016–2018) and thus requires further validation in different ethnic groups to ensure its generalisability. Ethnic/genetic factors (e.g., variations in metabolic profiles), healthcare system differences (e.g., access to follow-up care), and temporal changes (e.g., evolving revascularization techniques) might alter feature importance or performance; for instance, obesity metrics like abdominal circumference may vary by ethnicity. In conclusion, the retrospective nature of data collection at multiple centres across the country and across provinces resulted in a situation of missing data. While strict inclusion and exclusion criteria and large sample sizes may mitigate this limitation, prospective international multicentre studies are required to further validate the performance of the model. We recommend prospective multi-ethnic cohorts (e.g., including European/African-American populations) and temporal updates with recent data (post-2020) before clinical implementation, potentially via federated learning to assess cross-system applicability. Future research should incorporate prospective data, advanced omics, and real-time integration for enhanced precision.

In conclusion, our RF model provides a reliable, interpretable tool for identifying high-risk PRA patients, emphasizing novel predictors like depression and PT alongside established ones. This advances early risk stratification beyond traditional approaches, with potential to inform personalized treatment and follow-up strategies, ultimately optimizing management for this high-risk population.

## Data Availability

The raw data supporting the conclusions of this article will be made available by the authors, without undue reservation.
