# Supplementary material for: Development and validation of a machine learning predictive model for one-month post-revascularization angina in patients who had undergone PCI or CABG
Source: Front Cardiovasc Med. 2026 Feb 13;13:1747832. doi: 10.3389/fcvm.2026.1747832 (PMC12945824; doi:10.3389/fcvm.2026.1747832)
Supplement: Supplementary file 1 [file Table1.docx]

**SUPPLEMENTAL MATERIAL**

**Table S1. Information on clinical research centers, provinces, and regions**

| **Regions** | **Provinces** | | | **Clinical research centers** | **Encoding** |
| --- | --- | --- | --- | --- | --- |
| North China | Beijing | | | Beijing First Hospital of Integrated Chinese and Western Medicine | 0101 |
|  |  |  |  | Beijing Fengtai Hospital of Integrated Chinese and Western Medicine | 0102 |
|  |  |  |  | Xiyuan Hospital of China Academy of Chinese Medical Sciences (CACMS) | 0104 |
|  |  |  |  | Dongfang Hospital of Beijing University of Chinese Medicine | 0105 |
|  |  |  |  | Gaung’anmen Hospital of CACMS | 0106 |
|  |  |  |  | Beijing Chaoyang District Sanmafang Hospital | 0107 |
|  | Tianjin | | | First Teaching Hospital of Tianjin University of Traditional Chinese Medicine | 0201 |
|  |  |  |  | The Second Affiliated Hospital of Tianjin University of Traditional Chinese Medicine | 0202 |
|  | Hebei | | | School of Basic Medical Sciences, Hebei University of Chinese Medicine | 0301 |
|  |  |  |  | Hebei Provincial Hospital of Traditional Chinese Medicine | 0302 |
| Northeast China | Inner Mongolia | | | The Affiliated Hospital of Inner Mongolia Medical University | 0401 |
|  |  |  |  | People's Hospital of Inner Mongolia Autonomous Region | 0402 |
|  | Heilongjiang | | | The First Hospital Affiliated to Heilongjiang University of Chinese Medicine | 0501 |
|  | Jilin | | | Affiliated Hospital of Changchun University of Traditional Chinese Medicine | 0601 |
|  | Liaoning | | | Affiliated Hospital of Liaoning University of Traditional Chinese Medicine | 0701 |
|  |  |  |  | The Second Affiliated Hospital of Liaoning University of Traditional Chinese Medicine | 0702 |
| Middle East China | Shandong | | | Affiliated Hospital of Shandong University of Traditional Chinese Medicine | 0801 |
|  |  |  |  | Central Hospital Affiliated to Shandong First Medical University | 0802 |
|  | Shanghai | | | Shuguang Hospital Affiliated to Shanghai University of Traditional Chinese Medicine | 0901 |
|  | Zhejiang | | | Zhejiang Provincial Hospital of Traditional Chinese Medicine | 1001 |
|  |  |  |  | Zhejiang Provincial Tongde Hospital | 1002 |
|  | Henan | | | The First Affiliated Hospital of Henan University of Chinese Medicine | 1101 |
|  |  |  |  | Yudong Hospital, The First Affiliated Hospital of Henan University of Chinese Medicine | 1102 |
|  | Jiangsu | | | Affiliated Hospital of Integrated Traditional Chinese and Western Medicine, Nanjing University of Chinese Medicine | 1201 |
| Northwest China | | Shaanxi | Shaanxi Provincial Hospital of Traditional Chinese Medicine | | 1301 |
|  |  | Xinjiang | Xinjiang Uygur Autonomous Region Hospital of Traditional Chinese Medicine | | 1401 |
|  |  |  | Urumqi Hospital of Traditional Chinese Medicine | | 1402 |
|  |  | Ningxia | The Affiliated TCM Hospital of Ningxia Medical University | | 1501 |
|  |  |  | Yinchuan Hospital of Traditional Chinese Medicine Affiliated to Ningxia Medical University | | 1502 |
|  |  |  | Ningxia Institute of Traditional Chinese Medicine | | 1503 |
|  |  | Shanxi | Shanxi Provincial Hospital of Traditional Chinese Medicine | | 1601 |
|  |  |  | Affiliated Hospital of Shanxi University of Chinese Medicine | | 1602 |
| South China | | Yunnan | Kunming Hospital of Traditional Chinese Medicine | | 1801 |
|  |  | Hunan | The First Hospital of Hunan University of Chinese Medicine | | 1901 |
|  |  | Hubei | Hubei Provincial Hospital of Traditional Chinese Medicine | | 2001 |
|  |  |  | Xiangyang Hospital of Traditional Chinese Medicine | | 2002 |
|  |  |  | Huangshi Hospital of Traditional Chinese Medicine | | 2003 |
|  |  |  | Shiyan Combined Traditional Chinese and Western Medicine Hospital | | 2004 |
|  |  |  | Shiyan Taihe Hospital | | 2005 |
|  |  | Guangxi | The First Affiliated Hospital of Guangxi University of Chinese Medicine | | 2101 |
|  |  |  | Ruikang Hospital affiliated to Guangxi University of Chinese Medicine (Nanning Hospital of Traditional Chinese Medicine) | | 2102 |
|  |  | Guangdong | Guangdong Hospital of Traditional Chinese Medicine | | 2201 |
|  |  |  | The First Affiliated Hospital of Guangzhou University of Chinese Medicine | | 2202 |
|  |  |  | Maoming Hospital of Traditional Chinese Medicine | | 2203 |
|  |  | Fujian | Department of Cardiology I, The Affiliated People's Hospital of Fujian University of Traditional Chinese Medicine | | 2301 |
|  |  |  | Department of Cardiology II, The Affiliated People's Hospital of Fujian University of Traditional Chinese Medicine | | 2302 |
|  |  |  | The Second People's Hospital Affiliated to Fujian University of Traditional Chinese Medicine | | 2303 |
|  |  | Jiangxi | Jiangxi Provincial Hospital of Traditional Chinese Medicine | | 2401 |

**Supplemental Methods**

**Hyperparameter Optimization Protocol**

The hyperparameter tuning process for each machine learning algorithm was conducted as follows:

**Search Strategy**

1. Grid Search was employed for low-dimensional parameter spaces (Logistic Regression, KNN).
2. Random Search with 50 iterations used for tree-based models (RF, XGBoost, LightGBM).
3. Bayesian Optimization applied to Naive Bayes due to probabilistic parameter dependencies.

**Selection Criteria**

Optimal parameters were selected based on:

1. 5-fold CV AUROC maximization (primary criterion)
2. Early Stopping: Training halted if no AUROC improvement in 10 consecutive epochs
3. Complexity Penalty: Final model required <20% relative performance drop on validation vs training

**Table S2. Parameter Space Definitions**

| **Algorithm** | **Hyperparameters** | **Search Range** | **Tuning Method** |
| --- | --- | --- | --- |
| Logistic Regression | Penalty (L1/L2) C (regularization) | [0.001, 100] (log scale) | Grid Search (100-point log grid) |
| Random Forest | n_estimators max_depth min_samples_split | [50-500] [3-15] [2-10] | Random Search |
| Xgboost | learning_rate max_depth subsample | [0.01-0.3] [3-10] [0.6-1.0] | Bayesian (GP-EI) |
| LightGBM | num_leaves min_data_in_leaf feature_fraction | [16-256] [20-100] [0.6-1.0] | Random Search |
